# Poor oral health, related quality of life, and use of dental care system among vulnerable migrants in Denmark – a cross-sectional study

**DOI:** 10.2340/aos.v84.44658

**Published:** 2025-09-02

**Authors:** Josefina Salazar, Hanne Winther Frederiksen, Hanne Nødgaard Christensen, Esben Boeskov Øzhayat

**Affiliations:** a Department of Odontology, Faculty of Health and Medical Sciences, University of Copenhagen, Copenhagen, Denmark; b Section of Immigrant Medicine, Department of Infectious Diseases, University Hospital Copenhagen, Hvidovre, Denmark

**Keywords:** Oral health, quality of life, immigrants, dental health service

## Abstract

**Objective:**

The study aims to describe the oral status, oral health-related quality of life (OHRQoL), and oral-health behaviours of vulnerable adult migrants with complex health problems.

**Methods:**

We conducted a descriptive cross-sectional study at the Section of Immigrant Medicine, Hvidovre Hospital, Denmark. We conducted clinical examinations to assess participants’ number of teeth, caries, oral hygiene, and periodontal status. Participants also completed a questionnaire to evaluate OHRQoL, oral-health related behaviours (e.g. use of dental care system), and perceived barriers to access dental treatment and maintaining oral hygiene.

**Results:**

We included 50 participants, with a mean age of 49.4 (SD 7.4) years, 82% of whom were women, and 40% were from the Middle East and North Africa. Regarding oral status, 28% of participants had a full dentition, 54% had decayed teeth, 12% had good oral hygiene, and 46% had periodontal disease. Regarding OHRQoL, 92% of participants reported that their oral health negatively impacted one or more daily activities. Around 40% of participants had visited the dentist in Denmark the previous year, and 95% reported the costs as a barrier to dental care.

**Conclusions:**

There is a high prevalence of oral disease with a massive impact on the OHRQoL of this group of vulnerable migrants. Despite the need for essential dental treatments, participants rarely used the Danish dental care system. Our findings highlight the need for multi-sectoral interventions for this population.

## Introduction

Migrants in Europe involve a diverse group of people, such as economic migrants, asylum seekers, or displaced persons. They have different backgrounds and often have several distinct health-related challenges [1, 2] and further face barriers to accessing health and social services in the host country tied to the particular situation of each individual [3].

Migrants in Denmark have a higher prevalence of certain conditions, such as diabetes and poor mental health, and generally experience poorer health outcomes than their Danish counterparts [4, 5]. In addition, studies have shown that their oral health is also poor compared to the rest of the Danish population, as children and young people with a migrant background have an increased risk of oral diseases [6, 7], and adults with a non-western background are less likely to have a functional dentition than ethnic Danes [8, 9].

Oral health is integral to general health and well-being [10]. Oral diseases are associated with diabetes and cardiovascular disease [11–13] and pain from the mouth and teeth can even lead to inappropriate use of painkillers and sometimes addictive drugs [14]. Further, poor oral health affects quality of life [15] and can lead to social isolation [16]. Even though previous studies provide information about the increased risk of oral diseases among migrants in Denmark, there is a lack of reports about the oral status and oral health-related behaviours and impairments of those more vulnerable and socially marginalized. Conducting studies in a group of vulnerable migrants, that is those disadvantaged regarding socioeconomic conditions, language skills, and overall health status, is relevant since this group is at higher risk of experiencing more access barriers and being underserved in terms of health services [3].

Oral diseases are caused by a range of modifiable risk factors with the most obvious being high sugar consumption and poor oral hygiene, and their underlying social determinants, including low education, health beliefs, and the lack of availability of prevention and treatment services [17, 18]. Non-western migrants may hold different health beliefs compared to the Western population [3], and studies conducted in other countries have found that some groups give little importance to their oral health and oral hygiene practices [19–21]. Further, although most healthcare services are provided free of charge in Denmark, and certain hospitals have established migrant health clinics to increase health equity, the Danish dental care system primarily operates on a user-payment basis, and it is facilitated mainly through private practices. This is presumed to be a significant barrier to adequate access to care for socioeconomically vulnerable migrants [3, 18], but there is limited information about their actual experience.

The lack of evidence about the oral health and related behaviours for vulnerable migrants may be related to challenges in identifying, recruiting, and motivating participation in research, mainly because of cultural differences and a general lack of trust towards researchers [22, 23]. In this regard, a migrant health clinic provides an adequate setting to conduct a study on oral health and thus obtain relevant insights into a hard- to-reach group of vulnerable migrants often underrepresented in research.

We aimed to describe the oral status, oral health-related quality of life (OHRQoL), and oral-health behaviours, including the use of the dental care system, of vulnerable adult migrants with complex health problems. A better understanding of these aspects is needed to inform policymakers, healthcare providers, and other stakeholders working with migrants, to design and implement appropriate and coherent health services, ensuring better oral and general health and life for this population.

## Methods

This study is reported following the STROBE statement for cross-sectional studies [24].

### Participants

We conducted a cross-sectional study at the Section of Immigrant Medicine (SIM), Department of Infectious Diseases, Hvidovre Hospital, Capital Region, Denmark. SIM is a medical outpatient service assigned to secure examination, treatment, and rehabilitation of vulnerable patients with a migrant background, and sees approximately 150 patients annually. The referral criteria include (1) complex symptomatology, multimorbidity, or polypharmacy, often in combination with psychosocial stress factors, where (2) problems are assessed as not suitable for primary care or other services. This clinic has described their patients as more disadvantaged regarding socioeconomic background and language skills than other migrant populations, with complex patterns of symptoms and morbidity and a high prevalence of pain-related and musculoskeletal problems [25].

### Data collection

We collected data from November 2021 to February 2022. The doctor, nurse, or pharmacist at SIM recruited patients on the days that the research assistant was present at the clinic, which was twice a week. Recruiters informed the patients about the research project and obtained written informed consent from those who agreed to participate. We did not have any specific inclusion and exclusion criteria; however, recruitment of participants was left to SIM personnel’s discretion, generally depending on the patient’s overall health state and ability to cooperate.

### Measures

#### Oral health

We assessed oral health through a clinical examination and a questionnaire. All clinical examinations were performed by the research assistant (JS), a dentist, and they took place at SIM, using their standard equipment (regular chair and mobile surgical lamp), a basic exam kit (mirror, probe, tweezers, periodontal probe). During this examination, we recorded the number of teeth (including those with fixed prosthetics and excluding third molars), and the number of decayed teeth, that is teeth with cavitated caries.

We assessed oral hygiene using the simplified oral hygiene index (OHI-S) [26]. This index has two components, Debris Index and Calculus Index, each indicating the average scores for six pre-selected tooth surfaces. The values for the Debris Index and Calculus Index can range from 0 to 3, while the OHI-S values can range from 0 to 6, with higher values indicating poorer oral hygiene. We interpreted OHI-S scores as good (0–1.2), fair (1.3–3.0) and poor (3.1–6.0) oral hygiene [27].

We evaluated the periodontal status using the Community Periodontal Index of Treatment Needs (CPITN) [28] due to its simplicity and speed. It examines the presence or absence of common treatable conditions, namely periodontal pockets, calculus, and gingival bleeding in index teeth for each sextant, thus providing relevant information to guide care programs. Scores range from 0, indicating healthy periodontium, to 4, representing the presence of a periodontal pocket of 6 mm or more. We present the highest score recorded for each participant.

Participants completed a written questionnaire (Appendix 1) with the help of an interpreter through video conference. At SIM, the help of interpreters via video conference is routinely used, and patients are familiar with this procedure. The participants were also given the option to answer the questionnaire in their own home, provided they were proficient in any of the languages it was translated into: Arabic, Urdu, Turkish, and Danish, which are the most frequently spoken languages among the patients affiliated to SIM. The questionnaires were professionally translated and checked by personnel from SIM proficient in each of the languages.

We used the Oral Impact on Daily Performance (OIDP) scale to assess OHRQoL [29]. This comprises eight dimensions that could be impacted by problems with the mouth and teeth: (1) eating and enjoying foods, (2) speaking and pronouncing clearly, (3) cleaning teeth, (4) sleeping and relaxing, (5) smiling, laughing, and showing teeth without embarrassment, (6) maintaining usual emotional state without becoming irritable, (7) carrying out major work or playing a social role, (8) enjoying contact with other people. The possible answers are presented as the frequency in the last 6 months using a five point-Likert scale, ranging from never (0) to every or almost every day (4). Following the approach of previous studies [30], we dichotomized the answers for each dimension in absence of impact (answer 0) and the presence of impact (answers 1 to 4). From there, we calculated a summary score for each participant ranging from 0 to 8, indicating the number of dimensions impacted by their oral health.

In addition, we asked about participants’ overall satisfaction with their teeth, mouth, jaws, and dentures (if applicable), with the following response options: satisfied, dissatisfied, neither satisfied nor dissatisfied, and do not know. Lastly, we asked participants whether in the past 6 months they had experienced pain in their teeth, mouth, or jaws, or had felt their mouth dry, with possible response options being yes, no, or do not know for both questions.

#### Oral health behaviours

The use of the dental care system was assessed by asking about (1) number of dental visits within the last 12 months, (2) the place (country) of the visit, and (3) the reason for the last visit (pain/check-up/treatment). To present the results, we dichotomized the dental visits in use (one or more visits in the past 12 months) or no use. Additionally, we asked about nine potential barriers (see Appendix 1 and Table 3) in accessing dental treatment, with the question: ‘Do you think that the following situations could be a problem for you to get the dental care you want to receive?’ and the following response options: not a problem, somewhat of a problem, and major problem. We dichotomized the answers in problem (including the two latter categories) and not a problem.

We assessed oral hygiene habits with a question about the frequency of use of toothbrush and interproximal hygiene aids (including tooth floss and toothpicks). The possible responses were presented using a four point-Likert scale, ranging from never (0) to several times a day (3) [31]), and we dichotomized the results and present the number of participants who reported using these aids at least once a day (answers 2 and 3). Furthermore, we asked about the use of fluoride toothpaste (yes/no answer). We also asked about four potential barriers (see Appendix 1 and Table 3) to maintain oral hygiene and dichotomized the answers in problem and not a problem.

#### Sociodemographic information

Sociodemographic information was obtained from the registers of the clinic, and included age, sex, education, socioeconomic, and migration data, including nationality and date and grounds for residence permit. We present the results using the categorization recorded by the clinic.

### Analysis

We used descriptive analysis to characterize the sample and the variables assessed. We used medians and interquartile range (IQR) for quantitative variables and percentages for categorical variables. We conducted analyses using IBM SPSS statistics version 27. We did not attempt to perform inferential statistics due to the small sample size.

### Ethics

This study was registered in the local Data Protection register at the Faculty of Health Sciences, University of Copenhagen (514-0651/21-3000), which is approved by the Danish Data Protection Authority. Under the Danish law, this project does not need ethical approval, which was documented by the regional scientific ethics committee (Journal-no. 21056249).

Findings from the clinical examination were conveyed to each participant and when treatment was required, the participants were encouraged to see a dentist and were informed about the possibility of applying to the municipality for financial aid.

## Results

### Participants

We included a total of 50 participants, with a mean age of 49.4 (SD: 7.4) years, most of whom were women (82.0%), married (68.0%), and came mainly from the Middle East and North Africa region (40.0%) (Table 1). Around half of the participants had an education of less than 8 years (45.8%), and almost all received some sort of social subsidy (93.7%). Regarding migration, participants had received their residence permit in a median of 22 years ago (IQR: 10.5, 28.5), mostly through reunification to refugees or other migrants (68.0%). Most (72.0%) needed the assistance of an interpreter when attending their medical appointments. Forty-one participants answered the questionnaire, while all underwent the oral clinical examination. However, it was not possible to assess the CPITN in one case due to pain and extreme discomfort experienced by the participant.

**Table 1:** Sociodemographic characteristics of the included participants.

Characteristic	*n* (%)^a^
*Age,* mean (SD)	49.4 (7.4)
*Sex*	
Women	41 (82.0)
Men	9 (18.0)
*Civil Status*	
Married	34 (68.0)
Single	1 (2.0)
Divorced	15 (30.0)
*Source of basic income*	
Self-supported	3 (6.4)
Governmental subsidies	44 (93.7)
*Education*	
0–2 years	5 (10.4)
3–7 years	17 (35.4)
8–10 years	10 (20.8)
Vocational training	6 (12.5)
Short higher education (<2 years)	6 (12.5)
Medium and long higher education (>2 years)	4 (8.4)
*Region of origin (World Bank Classification)*	
Middle East and North Africa	20 (40.0)
Europe and Central Asia^b^	16 (32.0)
South Asia	10 (20.0)
Africa	3 (6.0)
Latin America and the Caribbean	1 (2.0)
*Residence Permit Background*	
Reunified to refugee	14 (28.0)
Reunified to other migrants	20 (40.0)
Refugee	13 (26.0)
Labour or study	3 (6.0)
*Time since residence permit,* median (IQR)	
<10	11 (22.4)
10–19	6 (12.2)
20–29	21 (42.9)
30+	11 (22.4)
*Need for Interpreter* ^c^	
No	14 (28.0)
Yes	36 (72.0)

a % based on number of respondents to each question

b Includes Turkey, Russia, and North Macedonia

c at doctor’s appointment. Combination of yes, always, and yes, sometimes

IQR: interquartile range.

### Oral health

Table 2 presents the results for oral health assessments. Fourteen (28.0%) participants had a full dentition, and one (2.0%) was completely edentulous. The median number of teeth was 26 (IQR: 24.0, 28.0), and for the 35 partially edentulous, the median number of missing teeth was 3 (IQR: 2.0, 6.0). Overall, 45 (90.0%) participants had 20 or more teeth. The median OHI-S score was 2.16 (IQR: 1.5, 2.8), and six (12%) participants had a good oral hygiene. Around half of participants had at least one code 3 or 4 in the CPITN (48.0%), indicating presence of periodontal pocket. Lastly, more than half of participants had decayed teeth, with a median of 3 (IQR: 1.0, 4.0).

**Table 2:** Oral health measures for the included participants.

Clinical Examination (*N* = 50)	*n* (%)	median (IQR)
*Number of Teeth*	50 (100)	26 (23.8, 28.0)
*Tooth decay (DT > 0)*	27 (54.0)	3 (1.0, 4.0)
*OHI-S*	49 (98.0)	2.16 (1.5, 2.8)
Good Oral Hygiene	6 (12.0)	
Fair Oral Hygiene	37 (74.0)	
Poor Oral Hygiene	6 (12.0)	
* Debris index*		1.33 (1.0, 1.7)
* Calculus index*		0.66 (0.5, 1.0)
*CPITN*		
0: healthy	0	
1: bleeding observed after probing	1 (2.0)	
2: calculus detected	24 (48)	
3: pocket 4–5mm	19 (38)	
4: pocket 6 mm or more	4 (8.0)	
9: not recorded	1 (2.0)	
X: excluded	1 (2.0)	
Self-reported (*N* = 41)	*n* (%)^a^	
*Oral Impact on Daily Performance (OIDP)*		
*Number of activities affected*		
0	3 (7.3)	
1	4 (9.8)	
2	6 (14.6)	
3	2 (4.9)	
4	6 (14.6)	
5	3 (7.3)	
6	4 (9.8)	
7	5 (12.2)	
8	8 (19.5)	
*Satisfied with oral health*		
Yes	6 (16.2)	
No	22 (59.5)	
Neither nor	7 (18.9)	
Does not know	2 (5.4)	
*Pain within the last 6 months*		
Yes	33 (82.5)	
No	6 (15.0)	
Does not know	1 (2.5)	
*Dry mouth within the last 6 months*		
Yes	30 (75.0)	
No	7 (17.5)	
Does not know	3 (7.5)	

OHI: oral hygiene index; CPITN: Community Periodontal Index of Treatment Needs; IQR; interquartile range.

a % based on number of respondents to each question.

**Figure 1:**
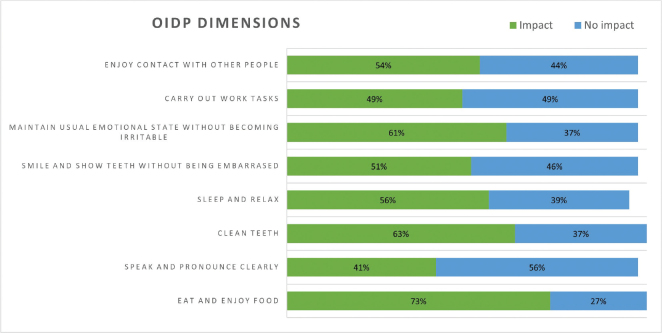
Frequency of impact of oral health on each dimension of the Oral Impact on Daily Performance (OIDP) scale in the past 6 months (N = 41).

Regarding the OIDP, three participants (7.3%) reported having zero impacts, and eight (19.5%) reported impacts on all dimensions. The median number of dimensions affected was four (IQR: 2.0, 7.0), and the dimensions that were more frequently affected were eating and enjoying food, cleaning teeth, and maintaining one’s usual emotional state (Figure 1). Additionally, most participants reported not being satisfied with their oral health (59.5%), having pain (82.5%), and feeling their mouth dry (75.0%) in the last 6 months.

### Oral health behaviours

Table 3 presents the results for oral health behaviours assessments. Around half of the participants reported having been to the dentist at least once in the last 12 months (55.0%). Among those who sought dental care, the main reason for consultation was pain, and 31.0% visited a dentist in a country other than Denmark. Participants reported encountering a median of 3 (IQR: 2.0, 4.0) barriers to accessing dental care, and the most frequently reported were the cost of dental treatment, lack of energy, and language difficulties.

**Table 3:** Oral health behaviours of the included participants (N = 41).

Oral health behaviour	*n* (%)^a^
Dentist visit within the last 12 months	
Yes	22 (55.0)
No	18 (45.0)
*Place of last dentist visit* ^*b*^	
Denmark	15 (68.1)
Other countries	5 (22.7)
*Reason for last dentist visit* ^*b*^	
Pain	16 (72.7)
Check-up/Treatment	5 (22.7)
Perceived access barriers	
Lack of knowledge about where to go	13 (34.2)
Language challenges	21 (52.5)
Costs	38 (95.0)
Lack of time	9 (23.1)
Dental fear or anxiety	9 (23.1)
Lack of energy	22 (56.4)
Difference in health beliefs with dentists	8 (21.6)
Being judged on appearance/ancestry/accent	12 (31.6)
Distance to the clinic	9 (23.1)
Oral Hygiene Habits	
*Use at least once a day*	
Toothbrush	33 (82.5)
Interproximal aids	12 (30.0)
*Use of fluoride toothpaste* ^*c*^	31 (79.5)
Perceived barriers to maintain oral hygiene	
Lack of knowledge	10 (25.6)
Lack of time	4 (10.8)
Costs of dental hygiene products	13 (34.2)
Lack of energy	16 (42.1)

a % based on number of respondents to each question

b % based on number of participants who answered that they have been to the dentist within the last 12 months

c number and % of participants that answered yes.

Regarding their oral hygiene habits, 33 participants (82.5%) reported brushing their teeth at least once a day, 26 (78.8%) of whom reported brushing several times a day. Thirty-one participants (79.5%) reported using fluoride toothpaste, and 15 (30.0%) reported using interproximal cleaning aids at least once a day. The most frequently reported barriers to maintaining oral hygiene were lack of energy (42.1%) and cost of dental hygiene products (34.2%).

## Discussion

The present study examined the oral health and oral health behaviours of a hard-to-reach group, consisting of adult migrants with complex health problems affiliated to a migrant health clinic. Our findings reveal a concerningly high prevalence of oral diseases and poor oral health, leading to high impact on quality of life and low satisfaction with oral health within this population. Despite the obvious need for essential dental treatments, the participants rarely used the Danish dental care system for regular controls, and unfortunately, so far this situation is unlikely to change, since participants reported facing numerous barriers for attending the system.

There are noticeable differences in the prevalence of oral disease between this group of migrants and the Danish population. The last epidemiological study on oral health in Denmark showed that among adults aged 45–54 years old, 99% had 20 or more teeth [32], compared to 90% in this group. Caries experience is also significantly lower among the Danish population, with a mean number of decayed teeth of 0.5 in adults aged 35–44 and 0.7 for adults who were 65–74 years old [33]. Lastly, regarding periodontal health, 41% in the 35–44 years old group had at least one code 3 or 4 using the CPITN [34] compared to 48% in this group. When comparing symptoms, 9.6–13.5% of Danes between 45 and 54 years old reported feeling dry mouth [32], compared to 75% in our study group, and 21% of Danes over 40 years old reported feeling pain in a 6-month period [35] compared to 82% in our study group.

In addition, the impact oral health status has on the OHRQoL in this population was very significant, with 92.7% of the participants reporting one or more daily activities being affected by their oral health. This number is higher than any found in previous studies using the OIDP. Compared to studies from Scandinavian countries, the difference is very pronounced, with 39.7% of older adults in Sweden [36], 28% of older adults in Norway [30], and 31.4 % of Danes over the age of 40 reporting any impact on daily activities [37]. When assessing the specific domains, previous studies have also found that eating and enjoying food is the most frequently affected activity [30, 35, 36, 38–41].

Regarding the utilization of dental care services by migrants, our findings are in agreement with other reports from the European region [42, 43]. A 2023 report from the National Institute of Public Health of Denmark revealed that the proportion of individuals that had regularly visited a dentist for a check-up at intervals of less than 12 months in the period 2017–2021 was 36.2% among migrants with a non-western background, compared to 55.7% for those with a Danish background [8]. Additionally, a report from the Danish Dental Association found that among ethnic Danes who rated themselves as having a low or very low social position, 45% did not go regularly to the dentist [35], which is similar to our findings in the group of migrants.

Similarly, several studies on migrant populations have also found that the high price of dental treatment is the main barrier to accessing dental care [44–46]. The Danish government offers subsidies for dental care through applications in the municipality to tackle financial difficulties. However, a recent study found that the uptake of this program has been very low, possibly due a lack of knowledge or information about this opportunity, or the highly bureaucratic process [47]. Hence, migrants continue to need targeted initiatives to overcome access barriers, considering that several reports from other countries showed that a majority of migrants, as opposed to a minority of non-migrants, reported difficulties in receiving dental treatment [44, 48].

Overall, the differences between the findings of our study and others may be explained by the unique characteristics of the population included in our study. Our study specifically focuses on a subset of the migrant population that is particularly vulnerable. Patients affiliated to SIM constitute a group with complex medical and psychosocial problems, often requiring physiotherapy and occupational therapy because of experiences with chronic pain [49], which can also explain the high proportion of participants reporting lack of energy as an access barrier and for maintaining their oral hygiene. Additionally, they generally have low levels of education and occupy low socio-economic positions, which exacerbate their challenges and represent key determinants for accessing healthcare services and their health-related behaviours [18].

It is also important to note that although many participants had resided in Denmark for over 20 years, the majority reported needing an interpreter when attending their medical appointments, and most perceived these language and cultural differences as barriers to accessing dental care. However, while several reports have studied acculturation as a determinant of oral health behaviours, focusing only on acculturation proxies such as language proficiency and length of stay may provide an incomplete explanation of migrants’ oral health behaviours and outcomes [50].

Our study has limitations that should be acknowledged. Caution is advised when generalizing our findings to other migrant populations, as we included a group of individuals that is particularly vulnerable. It is a cross-sectional study, which only allows to observe associations and not establish causation, however, the small sample size restricted our ability to conduct analyses. Nevertheless, it is worth highlighting that recruiting in hard-to-reach populations is a known challenge [51], and considering that they are underrepresented in research, we consider our results to be highly informative and to provide valuable insights. Additionally, there is a risk of selection bias since the inclusion of participants was carried out mainly by convenience sampling. This method may have led to the inclusion of those with a better overall condition, potentially underestimating the true magnitude of oral health problems faced by this population. However, it is worth noting that the demographic characteristics of the participants included in our study closely align with the population served by the clinic, as reported in a study by Rosenkrads and collaborators [25] based on 408 patients seen at SIM, suggesting that our sample is likely representative in that regard.

Another limitation is that the oral examination conditions were not optimal, and we did not have access to a dental chair, which may have led to a further underestimation of participants oral health problems. Lastly, there were language barriers, which we addressed by having interpreters and doctors to explain the questionnaire to the participants, and we further translated it into the most commonly spoken languages in the clinic. Still, there is a risk that some questions were misunderstood by participants.

Migrants’ health and health-related behaviours are to a large extent determined by the availability, accessibility, acceptability, and quality of health and social services in the host country [1]. The presence of migrant health clinics that address several health issues, but a lack of provision of dental care services, shows a lack of coherence and coordination across sectors. Considering the high prevalence of oral diseases in the population served by the migrant health clinic, it is essential to facilitate transitions between sectors and develop strategies to integrate dental care systems into the broader healthcare system effectively. Furthermore, although most participants reported brushing their teeth daily, only 12% had good oral hygiene, according to the clinical examination. Thus, tailored oral health promotion interventions may also be considered, as previous studies have found that these may result in a better ability to act in relation to one’s health and in positive oral hygiene self-care behaviours [52, 53].

There is a high prevalence of oral disease with a massive impact on the OHRQoL of this group of vulnerable migrants. In addition to providing relevant information about the oral health status of vulnerable migrants and highlighting its massive impact on their daily lives, our results offer insights into the primary access barriers encountered by this population. These findings can serve as a foundation for conducting further qualitative research, aimed at expanding our understanding of the experiences related to oral healthcare among this specific population, and can be used to inform policy makers in designing much needed interventions to address the inequities in oral health.

## Disclosure statement

The authors have no competing interests to declare.

## Author contribution statement

All authors contributed to the study conception and design. Material preparation, data collection, and analysis were performed by JS. The first draft of the manuscript was written by JS and all authors commented on previous versions of the manuscript. All authors read and approved the final manuscript.

## Data availability statement

Data are available from the corresponding author upon reasonable request.
